# Imaging the Bone-Immune Cell Interaction in Bone Destruction

**DOI:** 10.3389/fimmu.2019.00596

**Published:** 2019-03-26

**Authors:** Tetsuo Hasegawa, Junichi Kikuta, Masaru Ishii

**Affiliations:** ^1^Department of Immunology and Cell Biology, Graduate School of Medicine and Frontier Biosciences, Osaka University, Osaka, Japan; ^2^Division of Rheumatology, Department of Internal Medicine, Keio University School of Medicine, Tokyo, Japan; ^3^WPI-Immunology Frontier Research Center, Osaka University, Osaka, Japan

**Keywords:** intravital imaging, two-photon microscopy, cellular dynamics, bone, osteoclast, pH probe

## Abstract

Bone is a highly dynamic organ that is continuously being remodeled by the reciprocal interactions between bone and immune cells. We have originally established an advanced imaging system for visualizing the *in vivo* behavior of osteoclasts and their precursors in the bone marrow cavity using two-photon microscopy. Using this system, we found that the blood-enriched lipid mediator, sphingosine-1-phosphate, controlled the migratory behavior of osteoclast precursors. We also developed pH-sensing chemical fluorescent probes to detect localized acidification by bone-resorbing osteoclasts on the bone surface *in vivo*, and identified two distinct functional states of differentiated osteoclasts, “bone-resorptive” and “non-resorptive.” Here, we summarize our studies on the dynamics and functions of bone and immune cells within the bone marrow. We further discuss how our intravital imaging techniques can be applied to evaluate the mechanisms of action of biological agents in inflammatory bone destruction. Our intravital imaging techniques would be beneficial for studying the cellular dynamics in arthritic inflammation and bone destruction *in vivo* and would also be useful for evaluating novel therapies in animal models of bone-destroying diseases.

## Introduction

The interdisciplinary research field focusing on the crosstalk between the bone and immune systems, termed “osteoimmunology,” has revealed extensive reciprocal interplay between the two systems (1–3). Over the past two decades, a number of molecules, including cytokines, receptors, and transcription factors, have been shown to link the two systems, leading to successful translation of research into therapeutic approaches in osteoimmune diseases, such as rheumatoid arthritis (RA) (4). Bone and immune cells are in close contact with each other, and mechanisms of cell migration play a key role in their interplay. The development of an intravital imaging system using two-photon microscopy, combined with an increasing variety of fluorescent reporter mouse strains and fluorescence probes, has provided insight into the dynamic behavior of osteoclasts, osteoblasts, macrophages, and T cells in the bone marrow of living mice. This approach facilitates investigation of cellular dynamics in the pathogenesis of osteoimmune diseases and enables direct observation of complex biological phenomena *in vivo*. In this review, we discuss how the advances of imaging methods in living mice have contributed to our understanding of the bone–immune cell interaction in bone destruction. Furthermore, we introduce our recent studies, including evaluation of the interaction between osteoclasts and osteoblasts, and our novel approach for evaluating the mechanisms of action of different biological agents used for the treatment of bone-destructive diseases.

## Intravital two-photon Imaging of Bone Tissue

Bone is the hardest tissue in the body. It is technically difficult to visualize interactions between bone and immune cells in the bone marrow cavities of living animals. Although conventional methods such as micro-computed tomography, histomorphological analyses, and flow cytometry, can yield information on the bone structures and molecular expression patterns, *in vivo* information on dynamic cell movements and cellular interactions is not available (Table 1). Fluorescent microscopy imaging allows us to better understand the cellular dynamics of organs *in vivo* (5, 6), and we have established an imaging system to visualize living bone tissue using intravital two-photon microscopy (7–10).

**Table 1:** Comparison of different modalities used for bone research.

**Modalities**	**Advantages**	**Disadvantages**
Multi-photon microscopy (MPM)	√ Efficient light detection √ Reduced phototoxicity √ Penetrates deeper into tissues √ Detection of bone by second harmonic generation (SHG)	√ Higher cost √ Artifacts caused by autofluorescence √ Difficult to image more than four colors
Confocal microscopy	√ Easy to perform simultaneous, multicolor imaging √ High spatial and temporal resolutions	√ High phototoxicity and photobleaching √ Not suitable for thick tissues because of light scattering √ Artifacts caused by autofluorescence
MicroCT	√ Three-dimensional visualization of bone architecture √ Rapid	√ No cellular information √ No molecular information
Histochemistry	√ Inexpensive √ Highly specific for individual molecules	√ No vital cell information √ Enzymatic stains cannot be easily combined

Two-photon excitation-based laser microscopy affords several advantages compared to conventional confocal microscopy. In the latter technique, a fluorophore absorbs energy from a single photon and subsequently releases that energy as an emitted photon. In contrast, in the former technique, a fluorophore simultaneously absorbs two photons but only in the region of the focal plane where the photon density is high. Thus, all images are of high resolution. Second, excitation by a laser operating at near-infrared wavelength reduces phototoxic tissue damage, which is essential to yield reliable results. Third, light of near-infrared wavelengths penetrates deeper into tissue (to 100–1,000 μm) compared to confocal microscopy, which yields data to only a depth of <100 μm. Thus, two-photon excitation microscopy affords efficient light detection, reduces phototoxicity, and penetrates deeper into tissues, which makes it an important imaging tool for intravital visualization of the dynamic cellular behavior of deep tissues (5, 6).

Bone marrow is surrounded by calcium phosphate crystals of the bone matrix, which can readily scatter light of near-infrared wavelengths. However, in parietal bones of mice, the distance from the bone surface to the bone marrow cavity is only 80–120 μM, which is sufficiently thin to permit controlled fluorophore excitation within the cavity. Intravital two-photon imaging of skull bone tissue allows *in vivo* visualization of the real-time behavior of bone and immune cells in bone marrow cavities, such as osteoclasts, osteoblasts, macrophages, and lymphocytes. Moreover, such imaging may be useful when it is desirable to evaluate the effects of novel drugs targeting skeletal disease.

## Migratory Control of Osteoclast Precursors

**Figure 1:**
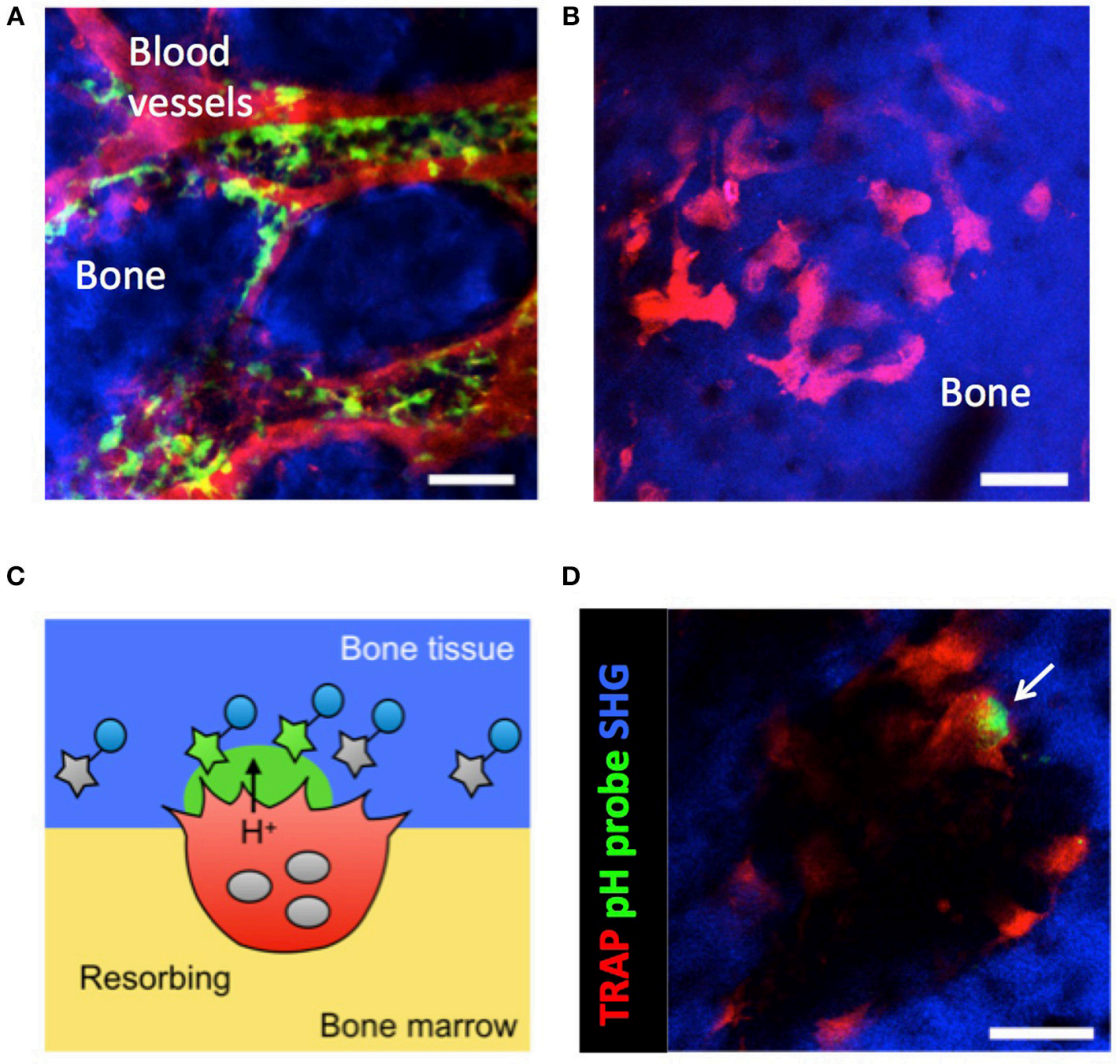
Intravital imaging of CX_3_CR1^+^ osteoclast precursors and mature osteoclasts in the bone marrow. **(A)** Image of the calvaria of CX_3_CR1-EGFP knock-in mice taken by two-photon microscopy. Osteoclast precursors are CX_3_CR1-EGFP^+^ (green). Blood vessels were stained via intravenous injection of Texas Red-conjugated dextran (red). Scale bar: 50 μm. The maximum intensity projections (MIPs) of two-dimensional image stacks of vertical calvarial slices are shown. **(B)** Images of the calvaria of TRAP-tdTomato transgenic mice taken by two-photon microscopy. Mature osteoclasts are tdTomato^+^ (red). Scale bar: 50 μm. Second harmonic fluorescence generated from two-photon excitation of collagen fibers defines the bone matrix (blue). The maximum intensity projections (MIPs) of two-dimensional image stacks of vertical calvarial slices are shown. **(C)** Schematic diagram of osteoclast localization and activity evaluation using a pH-sensing fluorescent probe. **(D)** Representative intravital two-photon images of the bone marrow of heterozygous TRAP-tdTomato transgenic mice treated with a pH-sensing fluorescent probe. Mature osteoclasts expressing TRAP-tdTomato signals (red), fluorescent signals from high H^+^ concentration (green), and second harmonic generation (SHG) defining the bone matrix. Some green fluorescent signal (arrow) could be detected along the bone surfaces near to osteoclasts. Scale bars: 50 μm. A two-dimensional image of the calvaria is shown.

Osteoclasts develop from cells of the monocyte/macrophage lineage. However, the means by which osteoclast precursor cells migrate to bony surfaces remain elusive. In previous work, intravital two-photon imaging of skull bone tissue allowed us to define the *in vivo* behavior of osteoclast precursor macrophages in the bone marrow (Figure 1A). We found that a blood-enriched mediator of lipid metabolism, sphingosine-1-phosphate (S1P), controlled the migratory behavior of osteoclast precursors in combination with several chemokines (7, 8).

S1P is a bioactive sphingolipid metabolite that regulates various biological activities, including cell proliferation, motility, and survival (11). S1P signaling is involved in T cell egress from lymphoid organs to circulatory fluids (12). Fingolimod (FTY720), a modulator of S1P receptor activity, was the first US Food and Drug Administration-approved oral therapy for relapsing forms of multiple sclerosis (MS) (13). S1P signaling involves five receptors, designated S1PR1 to S1PR5 (14, 15); osteoclast precursors in the bone marrow express both S1PR1 and S1PR2. S1PR1 is extremely sensitive to low S1P concentrations, promoting cell movement toward higher S1P concentrations in circulatory fluids, whereas S1PR2 requires a higher S1P concentration for activation and negatively regulates the S1PR1 response. When macrophages enter a low-S1P environment, such as the bone marrow, S1PR1 is transported to the cell surface and then osteoclast precursor macrophages move from bone tissue into the blood vessels, reflecting positive chemotaxis along an S1P gradient. Thus, the number of osteoclast precursor macrophages on bone surfaces is determined by bidirectional exchange of osteoclast precursors with the circulation. Many preclinical studies of S1P receptor modulators have been performed on autoimmune diseases (16), with an emphasis on the roles they play in inhibiting T cell migration, but their combined effects on cells of the monocyte/macrophage lineage require further exploration.

We have also showed that vitamin D controls the migratory behavior of osteoclast precursor macrophages by suppressing S1PR2 expression (10). In that study, intravital two-photon microscopy of bone marrow revealed that the motility of osteoclast precursor macrophages was significantly increased in mice treated with active vitamin D derivatives, suggesting that *in vivo* administration of active vitamin D suppresses both S1PR2 expression and mobilization of osteoclast precursor macrophages from the blood to the bone marrow. This results in suppression of osteoclastic bone resorption *in vivo* and it is the principal effect of active vitamin D. Thus, elucidation of the migratory behavior of osteoclast precursor macrophages to the bone surface has led to a better understanding of the mechanism of conventionally used medications.

## Regulation of Bone Resorbing Capacity of Mature Osteoclasts

Mature osteoclasts must be fluorescently labeled to allow their visualization by fluorescence microscopy. Fully differentiated osteoclasts form a tight attachment zone (a “sealing zone”) via interactions between integrin αvβ3 on the osteoclast membrane and bone matrix components (17). A number of vacuolar type H^+^-ATPases (V-ATPase) are specifically expressed along the ruffled border membrane to maintain highly acidic conditions in the resorption pit (18). V-ATPase is composed of multiple subunits, each of which has several isoforms. Of these, the a3 isoform of the a-subunit is preferentially and abundantly expressed in mature osteoclasts (19, 20). To fluorescently label mature osteoclasts, we generated mice expressing a3 subunit-GFP fusion proteins under the control of the original promoter of the a3 subunit (a3-GFP knock-in mice).

We also generated pH-sensing chemical fluorescent probes capable of detecting localized acidification by bone-resorbing osteoclasts on the bone surface *in vivo* (Figures 1C,D). These probes are based on the boron-dipyrromethene (BDPM) dye combined with a bisphosphonate group. BDPM dyes are used in several applications because of their environmental stability, large molar absorption coefficients, and high fluorescence quantum yields (21). The bisphosphonate group replaces the phosphate ion of hydroxyapatite (the principal component of bone tissue) to forms a tight bond with the bone matrix. Therefore, the bisphosphonate group facilitates probe delivery and fixation to bone in living animals (22). When mature osteoclasts secrete H^+^ for bone resorption, the probe detects the fall in local pH and emits a green fluorescent signal from the bone surface (9).

Our system that allows imaging of mature osteoclasts and bone-resorbing lesion *in vivo* via intravital two-photon microscopy has enabled us to identify two distinct functional states of osteoclasts; bone-resorbing (R) cells that are firmly adherent to bones and dissolve the bone matrix by secreting acids, and non-resorbing (N) cells that are relatively loosely attached to bones and moved laterally along bone surfaces (9). Treatment with recombinant RANKL, an essential osteoclastogenic cytokine under both homeostatic and arthritic conditions (23–28), changes the composition of these populations and the total number of mature osteoclasts. We have found that RANKL not only promotes osteoclast differentiation but also regulates the bone-resorptive function of fully differentiated mature osteoclasts (9).

Furthermore, CD4^+^ T helper 17 (Th17) cells, but not Th1, preferentially adhere to mature osteoclasts, although both T cell types migrate into bone marrow cavities to the same extent (9). Th17 cells express RANKL on the surface (29) and intravital bone imaging has shown that RANKL-bearing Th17 cells stimulate osteoclastic bone destruction by directly contacting N-state osteoclasts, converting such cells into the R-state (9). Pretreatment of Th17 cells with anti-RANKL neutralizing antibody or osteoprotegerin (OPG) reduces the interactions of such cells with the osteoclasts, but anti-RANKL antibody does not affect the mobility of Th1 cells. Thus, Th17 cells play a novel role, interacting with mature osteoclasts during inflammatory bone destruction.

## Crosstalk Between Osteoclasts and Osteoblasts

Bone is a dynamic tissue that undergoes continuous remodeling by bone-resorbing osteoclasts and bone-forming osteoblasts (30). Tight control of bone remodeling through a complex communication network between osteoblast and osteoclast lineage cells is critical for maintenance of bone homeostasis in response to structural and metabolic demands. In addition, the functional balance between these two cell types determines the final clinical manifestations of arthritic diseases, such as RA and psoriatic arthritis (PsA). In RA, pathological osteoclasts on the outer surface of the periarticular bone trigger devastating bone erosion, whereas PsA is characterized by inflammation of the connective tissue between tendon and bone, leading to new bone formation at enthesial sites created by osteoblasts. Therefore, it is essential to understand the spatiotemporal relationships and interactions between mature osteoblasts and osteoclasts *in vivo*.

**Figure 2:**
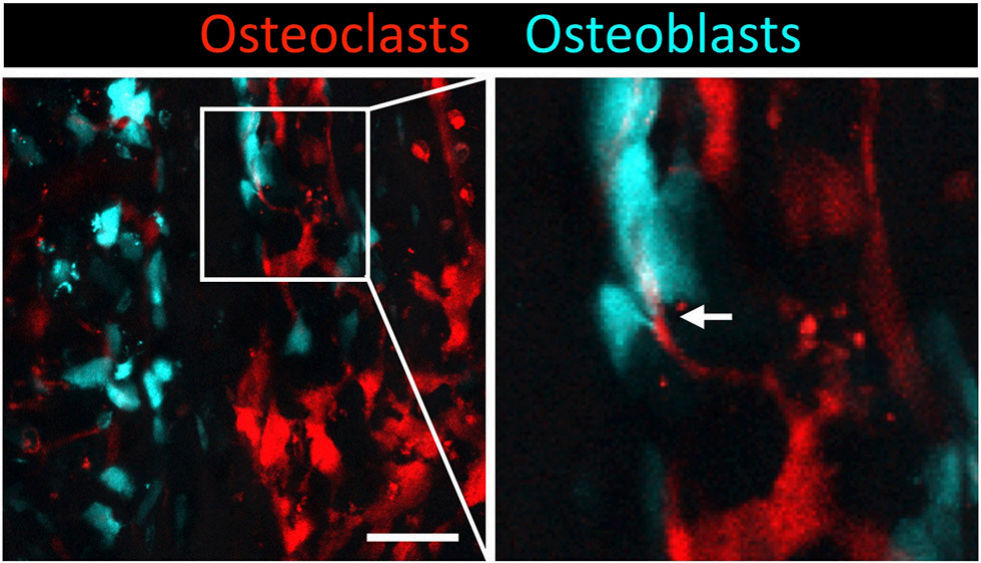
Intravital imaging of mature osteoclasts and osteoblasts in the bone marrow. Images of the calvaria of TRAP-tdTomato/Col2.3-ECFP double fluorescently labeled mice taken via two-photon microscopy. Scale bar: 50 μm. Maximum intensity projections (MIPs) of two-dimentional image stacks of vertical calvarial slices. Mature osteoclasts express TRAP-tdTomato signals (red) and mature osteoblasts express Col2.3-ECFP signals (cyan). Arrowhead indicates the direct osteoclast-osteoblast interaction.

To visualize mature osteoclasts, we generated transgenic reporter mice expressing tdTomato (a red fluorescent protein) in the cytosol of osteoclasts (TRAP-tdTomato mice) (Figure 1B) (9). To visualize mature osteoblasts, we recently generated mice expressing enhanced cyan fluorescent protein (ECFP) in the cytosol of osteoblasts (Col2.3-ECFP mice) (31). To visualize communications between osteoclasts and osteoblasts, we crossed TRAP-tdTomato mice with Col2.3-ECFP mice to generate TRAP-tdTomato/Col2.3-ECFP doubly fluorescent mice (Figure 2). Using intravital two-photon microscopy, we successfully visualized the *in vivo* behaviors of living osteoclasts and osteoblasts on the bone surface; imaging revealed direct interactions between osteoclasts and osteoblasts *in vivo*. In wide-field views of skull bones obtained under normal conditions, the osteoclasts and osteoblasts appeared to be separately distributed, although some direct osteoclast-osteoblast interactions were identified (Figure 2). Time-lapse images showed that several osteoclasts that were in contact with osteoblasts developed dendritic shapes and projected synapse-like structures toward the osteoblasts. Use of our imaging technique to visualize the osteoclasts and osteoblasts of animal models of arthritis may allow us to (at least in part) define why arthritis triggers osteolysis in certain disorders (such as RA) and osteogenesis in others (such as PsA).

In addition, a pH-sensing fluorescence probe revealed that osteoclasts secrete H^+^ for bone resorption when they are not in contact with osteoblasts, whereas osteoclasts in contact with osteoblasts are non-resorptive, suggesting that osteoblasts inhibit the bone resorption capacity of osteoclasts in a contact-dependent manner. Intermittent administration of parathyroid hormone led to a mixed distribution of osteoblasts and osteoclasts, thus increasing cell–cell contact to induce bone anabolic effects. The precise molecular mechanisms involved in the direct cell–cell contact should be explored in detail.

An earlier study used another mouse line featuring an osteoblast reporter, the Col2.3–GFP reporter line, to explore the interactions between T-cell acute leukemia and bone marrow microenvironment via two-photon microscopy (32). Further technical improvement in terms of bone marrow microenvironment imaging may reveal the detailed interplay between bone and the immune system not only in autoimmune diseases, but also in bone metastases and infectious diseases.

## Visualization of the Effects of Biological Agents on Macrophage Dynamics During Inflammatory Bone Destruction

Arthritic bone erosion in RA has been a major research topic in osteoimmunology. Works on the interplay between the immune and bone systems have suggested many useful drug development strategies. For example, proinflammatory cytokines, such as interleukin (IL) 6 and tumor necrosis factor α (TNFα), promote osteoclast differentiation by inducing RANKL in mesenchymal cells, and may directly stimulate both osteoclastogenesis and the bone-resorbing capacity of mature osteoclasts (33–37). Biological agents, such as monoclonal antibodies (mAbs) against IL-6 receptor (IL-6R) and TNFα, and CTLA4, have markedly improved the therapeutic outcomes of RA. Despite the differences in the molecular targets of these drugs, they equivalently suppress bone erosion in patients with RA and little is known about the differences in their modes of actions.

**Figure 3:**
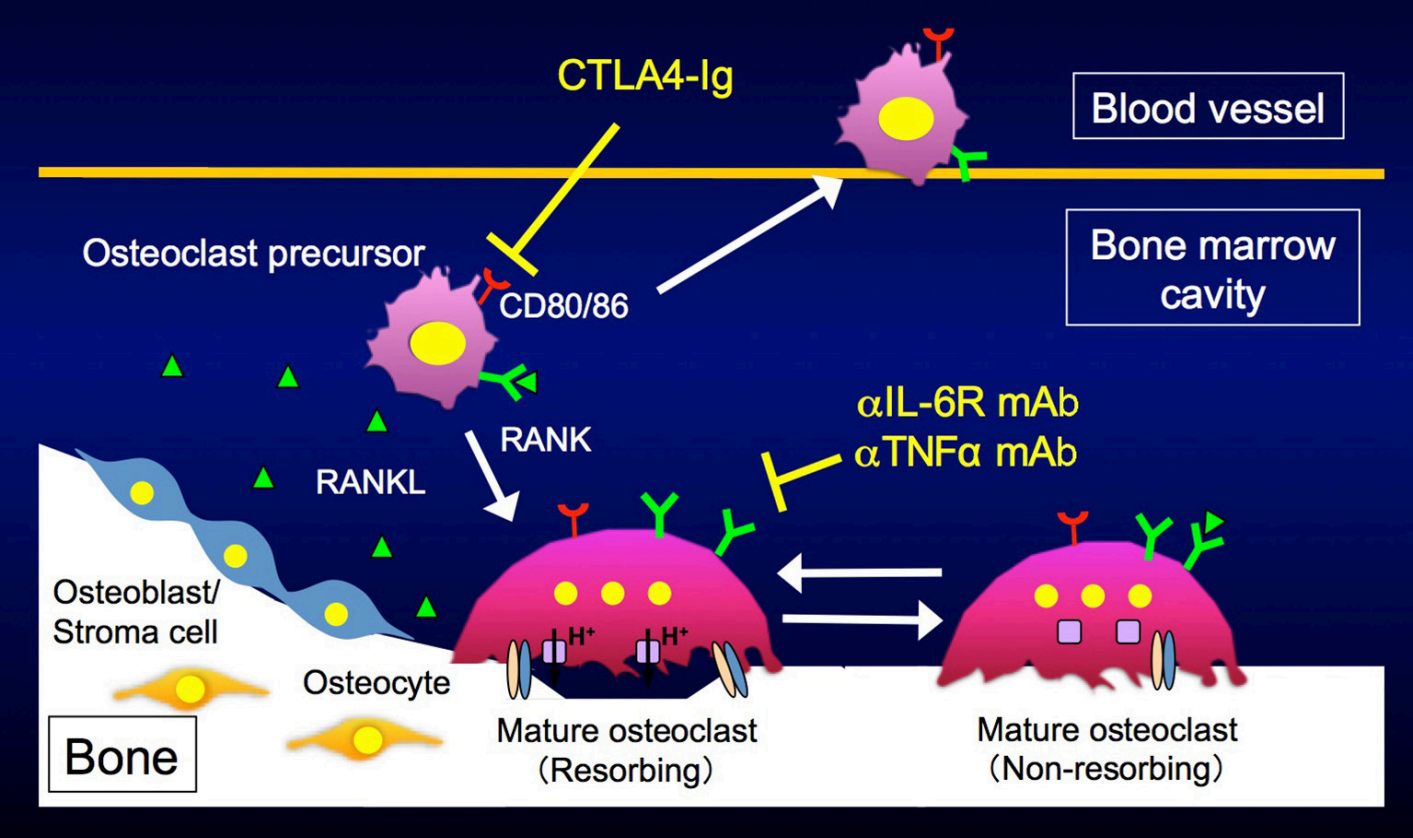
Different modes of action of biological disease-modifying antirheumatic drugs (DMARDs). Anti-IL6R and anti-TNFα monoclonal antibodies affect mature osteoclasts and switch bone-resorbing osteoclasts to non-resorbing cells. CTLA4 mobilizes osteoclast precursors, eliminating their attachment to bone surfaces.

Using the LPS injection model, we directly visualized the *in vivo* behavior of mature osteoclasts and their precursors during inflammatory bone destruction, and explored how different biological agents affect the dynamics of these cells *in vivo* (38). We found that anti-IL-6R and anti-TNFα mAbs affected mature osteoclasts and switched bone-resorbing osteoclasts to non-resorbing cells. On the other hand, CTLA4 had no effect on mature osteoclasts but mobilized osteoclast precursor macrophages, eliminating the firm attachment of such cells to bone surfaces. In agreement with these results, CD80/86, the target molecules of CTLA4, were prominently expressed in osteoclast precursor macrophages, but were suppressed during osteoclast maturation (Figure 3). Taken together, these data indicate that various biological agents acted at specific therapeutic points in states of inflammatory bone destruction, and these new findings may enable us to optimize treatment efficacy for each patient by adjusting therapeutic regimens and doses, representing an important step toward personalized medicine. The development of intravital bone imaging techniques for other inflammatory bone destruction models, such as collagen-induced arthritis, will allow us to better understand the modes of action of biologics within arthritic joints.

In addition, macrophages of osteal tissues are reported to be involved in the regulation of osteoblast function, and subsequently bone dynamics (39). The additive role played of CTLA4 in bone remodeling through mobilizing osteal tissue macrophages should be further examined in the future.

## Conclusion

Considerable progress has been made in clarifying the interplay between bone and immune cells under both physiological and inflammatory conditions. However, their dynamic crosstalk within living animals is still largely obscure. Intravital two-photon imaging provides unbiased spatiotemporal information on the biological phenomena in living organisms, which are often much more complex than we may have hypothesized. Therefore, it is important to incorporate technical developments in imaging, such as two-photon microscopy, to directly observe the biological phenomena *in vivo* and determine the precise interplay between bone and immune systems in future studies.

## Author Contributions

TH, JK, and MI contributed to the discussion and wrote and reviewed the manuscript.

### Conflict of Interest Statement

The authors declare that the research was conducted in the absence of any commercial or financial relationships that could be construed as a potential conflict of interest.
